# Boundedness of a class of rough maximal functions

**DOI:** 10.1186/s13660-018-1900-y

**Published:** 2018-11-09

**Authors:** Mohammed Ali, Omar Al-mohammed

**Affiliations:** Department of Mathematics and Statistics, Jordan University of Science and Technology, Irbid, Jordan

**Keywords:** 42B20, 40B25, 47G10, Maximal functions, $L^{p}$ boundedness, Rough kernels, Surfaces of revolution, Extrapolation

## Abstract

In this work, we obtain appropriate sharp bounds for a certain class of maximal operators along surfaces of revolution with kernels in $L^{q}(\mathbf{S}^{n-1})$, $q > 1$. By using these bounds and using an extrapolation argument, we establish the $L^{p}$ boundedness of the maximal operators when their kernels are in $L(\log L)^{\alpha}(\mathbf{S}^{n-1})$ or in the block space $B^{0,\alpha-1}_{q} (\mathbf{S}^{n-1})$. Our main results represent significant improvements as well as natural extensions of what was known previously.

## Introduction and main results

Throughout this article, let $\mathbf{R}^{n}$, $n\geq2$, be the *n*-dimensional Euclidean space and $\mathbf{S}^{n-1}$ be the unit sphere in $\mathbf{R}^{n}$ equipped with the normalized Lebesgue surface measure $d\sigma= d\sigma(\cdot)$. Also, let $x^{\prime}= x/|x|$ for $x\in\mathbf{R} ^{n} \setminus\{0\}$ and $p^{\prime}$ denote the exponent conjugate to *p*; that is, $1/p+1/p'=1$.

Let $K_{\varOmega,h}(y)=\varOmega(y)h( \vert y \vert ) \vert y \vert ^{-n}$, where $h:[0,\infty)\rightarrow\mathbf{C}$ is a measurable function and *Ω* is a homogeneous function of degree zero on $\mathbf{R}^{n}$ that is integrable on $\mathbf{S}^{n-1}$ and satisfies the cancelation property

1.1 $$ \int_{\mathbf{S}^{n-1}}\varOmega\bigl(x^{\prime}\bigr) \,d\sigma \bigl(x^{\prime}\bigr) =0. $$

For $1\leq\gamma<\infty$, define $\mathfrak{L}^{\gamma}(\textbf{R}^{+})$ to be the set of all measurable functions $h:\textbf{R}^{+}\rightarrow\textbf{R}$ that satisfy the condition $\Vert h \Vert _{L^{\gamma}( \mathbf{R} ^{+},\frac{dr}{r})}= (\int_{0}^{\infty} \vert h(r) \vert ^{\gamma}\,\frac{dr}{r} )^{1/\gamma}\leq1 $, and define $\mathfrak{L}^{\infty}(\textbf{R}^{+})={L^{\infty}( \mathbf{R} ^{+},\frac{dr}{r})}$.

For a suitable mapping $\phi: \textbf{R}^{+}\rightarrow\textbf{R}$, we define the maximal operator $\mathcal{M}_{P,\varOmega, \phi}^{(\gamma)}$ for $f \in\mathcal{S}(\mathbf{R} ^{n+1})$ by

1.2 $$ \mathcal{M}_{P,\varOmega, \phi}^{(\gamma)}(f) (x,x+1)=\sup_{h\in \mathfrak{L}^{\gamma}(\textbf{R}^{+}) } \biggl\vert \int_{ \mathbf{R} ^{n}}e^{i P(y)}f\bigl(x-y,x_{n+1}-\phi\bigl( \vert y \vert \bigr)\bigr)K_{\varOmega,h}(y)\,dy \biggr\vert , $$

where $P:\textbf{R}^{n}\rightarrow\textbf{R}$ is a real-valued polynomial.

When $P(y)=0$, we denote $\mathcal{M}_{P,\varOmega, \phi}^{(\gamma)}$ by $\mathcal{M}_{\varOmega, \phi}^{(\gamma)}$. Also, when $\phi(t)=t$, we denote $\mathcal{M}_{\varOmega, \phi}^{(\gamma)}$ by $\mathcal{M}_{\varOmega}^{(\gamma)}$ which is the classical maximal operator that was introduced by Chen and Lim in [17]. The authors of [17] proved that when $\varOmega\in\mathcal{C( \mathbf{S}}^{n-1}\mathcal{)}$ and $h\in\mathfrak{L}^{\gamma}( \mathbf{R} ^{+} )$ for some $1\leq\gamma\leq2$, then the $L^{p}$ boundedness of $\mathcal{M}_{\varOmega}^{(\gamma)}$ is satisfied for $(n\gamma)'< p<\infty$. This result was improved by Al-Salman in [10]; he established the $L^{p}(\mathbf{ \mathbf{R} }^{n})$ boundedness of $\mathcal{M}_{\varOmega}^{(2)}$ for all $p\geq 2$ provided that $\varOmega\in L(\log L)^{1/2}(\mathcal{\mathbf{S}}^{n-1})$. Moreover, he pointed out that the condition $\varOmega\in L(\log L)^{1/2}(\mathcal{\mathbf{S}}^{n-1})$ is optimal in the sense that $1/2$ in $L(\log L)^{1/2}(\mathcal{\mathbf{S}}^{n-1})$ cannot be replaced by any smaller positive number. In addition, the last result was generalized by Al-Qassem (see [4, Theorem 1.5]). Indeed, he verified that $\mathcal{M}_{\varOmega}^{(\gamma)}$ is bounded on $L^{p}(\mathcal{\mathbf{R}}^{n})$ for all $p\geq\gamma'$ and $1<\gamma\leq2$ under the condition $\varOmega\in L(\log L)^{1/{\gamma'}}(\mathcal{\mathbf{S}}^{n-1})$. Later on, Al-Qassem in [4] improved the above results. Precisely, he obtained that if $h\in\mathfrak{L}^{\gamma}(\textbf{R}^{+})$ for some $1\leq\gamma\leq2$, $\varOmega\in L(\log L)^{1/\gamma'}(\mathcal{\mathbf{S}}^{n-1})$; and *ϕ* is $C^{2}([0,\infty))$, convex and increasing function with $\phi(0)=0$, then $\mathcal{M}_{\varOmega, \phi}^{(\gamma)}$ is bounded on $L^{p}(\mathbf{ \mathbf{R} }^{n+1})$ for any $\gamma'\leq p<\infty$ with $1<\gamma\leq2$; and it is bounded on $L^{\infty}(\mathbf{ \mathbf{R} }^{n+1})$ for $\gamma=1$. On the other hand, when *Ω* belongs to the block spaces $B_{q}^{(0,-1/2)}(\mathbf{S}^{n-1})$ for some $q>1$, then the author of [3] showed that $\mathcal{M}_{\varOmega}^{(2)}$ is bounded on $L^{p}(\mathbf{ \mathbf{R} }^{n})$ for all $p\geq2$. Furthermore, he found *Ω* which lies in $B_{q}^{(0,-1/2-\varepsilon)}(\mathbf{S}^{n-1})$ for all $\varepsilon >0$ such that $\mathcal{M}_{\varOmega}^{(2)}$ in not bounded on $L^{2}(\mathcal{\mathbf{R}}^{n})$. Subsequently, the study of the $L^{p}$ boundedness of $\mathcal{M}_{\varOmega}^{(\gamma)}$ under various conditions on the function has been performed by many authors. The readers can see [9, 12, 20, 21, 23–25], and [28] for the significance of considering integral operators with oscillating kernels.

We point out that the study the maximal operator $\mathcal{M}_{P,\varOmega, \phi}^{(\gamma)}$ was initiated by Al-Salman in his work in [11]. In fact, he investigated the $L^{p}$ ($p\geq2$) boundedness of $\mathcal{M}_{P,\varOmega, t}^{(2)}$ under the condition $\varOmega\in L(\log L)^{1/2}(\mathcal{ \mathbf{S}}^{n-1})\cup B_{q}^{(0,-1/2)}( \mathcal{\mathbf{S}}^{n-1})$ for some $q>1$. For more information about the importance and the recent advances on the study of such operators, the readers are referred to [1, 2, 5, 27], and the references therein.

In view of the results in [4] as well as the results in [11], it is natural to ask whether the parametric maximal operator $\mathcal{M}_{P,\varOmega ,\phi}^{(\gamma)}$ is bounded on $L^{p}( \mathbf{R} ^{n+1})$ under weak conditions on *Ω*, *ϕ*, and *γ*. We shall obtain an answer to this question in the affirmative as described in the next theorem. Precisely, we will establish the following result.

### Theorem 1.1

*Suppose that* $\varOmega\in L^{q}(\mathbf{S} ^{n-1})$, $q> 1$, *and satisfy condition* (1.1) *with* $\Vert \varOmega \Vert _{L^{1}(\mathbf{S}^{n-1})}\leq1$. *Suppose also that* $\phi:\mathbf{R^{+}}\rightarrow\mathbf{R}$ *is in* $C^{2}([0,\infty))$, *convex and increasing function with* $\phi(0)=0$. *Let* $P:\mathbf{R}^{n}\rightarrow\mathbf{R}$ *be a polynomial of degree* *m* *and* $\mathcal{M}_{P,\varOmega,\phi}^{(\gamma)}$ *be given by* (1.2). *Then there exists a constant* $C_{p,q}>0$ *such that*

1.3 $$ \bigl\Vert \mathcal{M}_{P,\varOmega,\phi}^{(\gamma)}(f) \bigr\Vert _{L^{p}( \mathbf{R} ^{n+1})}\leq C_{p,q} (1+\beta_{\varOmega}) )^{1/\gamma'} \Vert f \Vert _{L^{p}( \mathbf{R} ^{n+1})} $$

*for* $\gamma'\leq p<\infty$ *and* $1<\gamma\leq2$; *and*

1.4 $$ \bigl\Vert \mathcal{M}_{P,\varOmega,\phi}^{(1)}(f) \bigr\Vert _{L^{\infty}( \mathbf{R} ^{n+1})}\leq C \Vert f \Vert _{L^{\infty}( \mathbf{R} ^{n+1})}, $$

*where* $\beta_{\varOmega}=\log(e+ \Vert \varOmega \Vert _{L^{q}(\mathbf{S}^{n-1})})$, $C_{p,q}=\frac{2^{1/q'}}{2^{1/q'}-1}C_{p}$, *and* $C_{p}$ *is a positive constant that may depend on the degree of the polynomial* *P* *but it is independent of* *Ω*, *ϕ*, *q*, *and the coefficients of the polynomial* *P*.

By the conclusion from Theorem 1.1 and applying an extrapolation argument (see [8, 11] and [26]), we get the following.

### Theorem 1.2

*Suppose that* *Ω* *is given as in Theorem *1.1 *and* $\mathcal{M}_{P,\varOmega,\phi}^{(\gamma)}$ *is given by* (1.2), *where* *ϕ* *is in* $C^{2}([0,\infty))$, *convex and increasing function with* $\phi(0)=0$. *If* $\varOmega\in L(\log L)^{1/\gamma'}(\mathcal{ \mathbf{S}}^{n-1})\cup B_{q}^{(0,-1/\gamma)}( \mathcal{\mathbf{S}}^{n-1})$, *then* $\mathcal{M}_{P,\varOmega ,\phi}^{(\gamma)}$ *is bounded on* $L^{p}(\textbf{R}^{n+1})$ *for* $\gamma'\leq p<\infty$ *and* $1<\gamma\leq2$; *and it is bounded on* $L^{\infty}(\textbf{R}^{n+1})$ *for* $\gamma=1$.

Here and henceforth, the letter *C* denotes a bounded positive constant that may vary at each occurrence but is independent of the essential variables.

## Preliminary lemmas

This section is devoted to present and prove some auxiliary lemmas which will be used in the proof of Theorem 1.1. We start with the following lemma which can be derived by applying the arguments (with only minor modifications) used in [11].

### Lemma 2.1

*Let* $\varOmega\in L^{q}(\mathbf{S}^{n-1})$, $q> 1$, *and satisfy condition* (1.1) *with* $\Vert \varOmega \Vert _{L^{1}(\mathbf{S}^{n-1})}\leq1$. *Assume that* $\phi(\cdot)$ *is an arbitrary function on* $\mathbf{R}^{+}$, *and assume also that* $P=\sum_{ \vert \alpha \vert \leq m}a_{\alpha}x^{\alpha}$ *is a polynomial of degree* $m\geq1$ *such that* $\vert x \vert ^{m}$ *is not one of its terms and* $\sum_{ \vert \alpha \vert = m} \vert a_{\alpha} \vert =1$. *For* $k\in \mathbf{Z} $, *define* $\mathcal{J}_{k,\varOmega,\phi}:\mathbf{R} ^{n+1}\rightarrow\mathbf{R} $ *by*

2.1 $$ \mathcal{J}_{k,\varOmega, \phi}(\xi,\eta)= \int _{1}^{2^{2\beta _{\varOmega}}} \biggl\vert \int_{\mathbf {S}^{n-1}}\varOmega (u)\mathcal{G}_{k,\varOmega,\phi}(r,u,\xi\cdot u, \eta)\,d\sigma (u) \biggr\vert ^{2}\,\frac{dr}{r}, $$

*where*

2.2 $$ \mathcal{G}_{k,\varOmega,\phi}(r,u,\xi\cdot u,\eta)=e^{-i [ P(2^{-(k+1)\beta_{\varOmega}}r u) + (2^{-(k+1)\beta_{\varOmega}})r u\cdot\xi+\phi(2^{-(k+1)\beta_{\varOmega}}r)\eta ] }. $$

*Then a positive constant* *C* *exists such that*

$$ \sup_{(\xi,\eta) \in\mathbf{R} ^{n}\times\mathbf{R} }\mathcal{J}_{k,\varOmega,\phi} (\xi,\eta)\leq C \beta_{\varOmega} 2^{{(k+1)/4q'}}. $$

### Proof

On the one hand, it is clear that

2.3 $$\begin{aligned} \mathcal{J}_{k,\varOmega,\phi}(\xi,\eta) \leq &C \int_{1}^{2^{2\beta_{\varOmega}}} \biggl( \int_{ \mathbf{S}^{n-1}} \bigl\vert \varOmega(u) \bigr\vert \,d\sigma(u) \biggr) ^{2}\,\frac{dr}{r} \leq C\beta_{\varOmega} \Vert \varOmega \Vert _{{L^{1}(\mathbf{S}^{n-1})}}^{2}\leq C\beta _{\varOmega}. \end{aligned}$$

Also, it is easy to get that

$$\begin{aligned} &P\bigl(2^{-(k+1)\beta_{\varOmega}}r u\bigr)- P\bigl(2^{-(k+1)\beta_{\varOmega}}r v\bigr) + 2^{-(k+1)\beta_{\varOmega}}r u\cdot \xi-2^{-(k+1)\beta_{\varOmega}}r v\cdot\xi \\ &\quad =2^{-m(k+1)\beta_{\varOmega}}r^{m} \biggl( \sum_{ \vert \alpha \vert =m} a_{\alpha}u^{\alpha}- \sum_{ \vert \alpha \vert =m} a_{\alpha}v^{\alpha}\biggr)+2^{-(k+1)\beta_{\varOmega}}r (u-v)\cdot \xi+A_{k}(u,v,r,\xi), \end{aligned}$$

with $\frac{d^{m}}{dr^{m}}A_{k}(u,v,r,\xi)=0$. Without loss of generality, we may assume that $m>1$. Then, we follow the same steps as in [11, (2.9)–(2.12)] to prove that the inequality

2.4 $$ \mathcal{J}_{k,\varOmega,\phi} (\xi,\eta)\leq C \beta ^{1-1/4q'}_{\varOmega} \bigl(2^{{(k+1)\beta_{\varOmega}/4q'}} \bigr) $$

holds for some constant $C>0$. Therefore, combining (2.4) with the trivial estimate (2.3) leads to

$$ \sup_{(\xi,\eta) \in\mathbf{R} ^{n}\times\mathbf{R} }\mathcal{J}_{k,\varOmega,\phi} (\xi,\eta)\leq C \beta_{\varOmega} 2^{{(k+1)/4q'}}. $$

 □

We shall need the following lemma which can be acquired by using the argument employed in the proof of [14, Lemma 4.7].

### Lemma 2.2

*Let* $\varOmega\in L^{1}(\mathbf{S}^{n-1})$ *be a homogeneous function of degree zero and satisfy condition* (1.1). *Suppose that* $\phi:\mathbf{R^{+}}\rightarrow \mathbf{R}$ *is in* $C^{2}([0,\infty))$, *convex and increasing function with* $\phi(0)=0$. *Let the maximal function* ${{\mathcal{M}}}_{\varOmega,\phi} $ *be given by*

$$ {{\mathcal{M}}}_{\varOmega,\phi} f(x,x_{n+1})=\sup_{\mathbf {j\in \mathbf{Z} }} \int_{2^{j}\beta_{\varOmega}\leq \vert y \vert \leq2^{j+1}\beta_{\varOmega}} \bigl\vert f\bigl(x-y,x_{n+1}-\phi\bigl( \vert y \vert \bigr)\bigr) \bigr\vert \frac{ \vert \varOmega(y) \vert }{ \vert y \vert ^{n}} \,dy. $$

*Then*, *for* $1< p\leq\infty$, *there exists a positive number* $C_{p}$ *so that*

$$ \bigl\Vert {{\mathcal{M}}}_{\varOmega,\phi} (f) \bigr\Vert _{L^{p}(\mathbf{ \mathbf{R} }^{n+1})} \leq C_{p} (1+\beta_{\varOmega })^{1/2} \Vert f \Vert _{L^{p}(\mathbf{ \mathbf{R} }^{n+1})} \Vert \varOmega \Vert _{L^{1}(\mathbf{S}^{n-1})} $$

*for every* $f\in L^{p}(\mathbf{ \mathbf{R} }^{n+1})$.

Using a similar argument as in the proof of [4, Theorem 1.6], we obtain the following.

### Lemma 2.3

*Let* $\varOmega\in L^{q}(\mathbf{S} ^{n-1})$, $q> 1$, *and satisfy condition* (1.1) *with* $\Vert \varOmega \Vert _{L^{1}(\mathbf{S}^{n-1})}\leq1$. *Assume that* $\phi(\cdot)$ *is given as in Theorem *1.1. *Then there exists a constant* $C_{p,q}>0$ *such that*

2.5 $$ \bigl\Vert \mathcal{M}_{0,\varOmega,\phi}^{(2)}(f) \bigr\Vert _{L^{p}( \mathbf{R} ^{n+1})}\leq C_{p,q} (1+ \beta_{\varOmega } )^{1/2} \Vert f \Vert _{L^{p}( \mathbf{R} ^{n+1})} $$

*for* $2\leq p<\infty$.

### Proof

Since $L^{q} ( \mathbf{S}^{n-1} ) \subseteq L^{2} ( \mathbf{S}^{n-1} )$ for $q\geq2$, it is enough to prove this lemma for $1< q\leq2$. It is clear that

$$\begin{aligned} \mathcal{M}_{\varOmega, \phi}^{(2)}(f) (x,x+1) =&\mathcal{M}_{0,\varOmega, \phi}^{(2)}(f) (x,x+1)\\ \leq& \biggl( \int_{0}^{\infty } \biggl\vert \int_{\mathbf{S}^{n-1}}f\bigl(x-r u,x_{n+1}-\phi(r)\bigr)\varOmega(u)\,d \sigma(u) \biggr\vert ^{2}\,\frac{dr}{r} \biggr) ^{1/2}. \end{aligned}$$

Let $\{ \varphi_{k} \}_{k\in \mathbf{Z}} $ be a smooth partition of unity in $(0,\infty)$ adapted to $\mathcal{I}_{k,\beta_{\varOmega}}= [ 2^{-(k+1)\beta _{\varOmega}},2^{-(k-1)\beta_{\varOmega}} ]$. More precisely, we require the following:

$$\begin{aligned} &\varphi_{k}\in\mathcal{C}^{\infty},\quad \operatorname{supp} \varphi_{k} \subseteq\mathcal{I}_{k,\beta_{\varOmega}},\quad 0\leq\varphi _{k}\leq1, \\ &\sum_{k\in\mathbf{Z}}\varphi_{k} ( r ) =1,\quad \mbox{and}\quad \biggl\vert \,\frac{d^{k}\varphi_{k} ( r ) }{dr^{k}} \biggr\vert \leq\frac{C_{k}}{r^{k}}. \end{aligned}$$

Define the multiplier operators $S_{k}$ in $\textbf{R}^{n+1}$ by

$$\begin{aligned} \widehat{{(S_{k}f)}}(\xi,\eta)=\varphi_{k}\bigl( \vert \xi \vert \bigr))\widehat{f}(\xi,\eta)\quad \mbox{for }(\xi,\eta)\in \mathbf{R}^{n}\times\mathbf{R}. \end{aligned}$$

Hence, for $f\in\mathcal{S}(\mathbf{R}^{n+1})$, we have

2.6 $$ \mathcal{M}^{(2)}_{0,\varOmega, \phi}(f) (x,x_{n+1})\leq \sum _{j\in \mathbf{Z}}\mathcal{T}_{\varOmega,\phi,j}f(x,x_{n+1}), $$

where

$$\begin{aligned} &\mathcal{T}_{\varOmega,\phi,j}f(x,x_{n+1})= \biggl( \int_{0}^{\infty} \bigl\vert \mathcal{N}_{\varOmega,\phi,j}(x,x_{n+1}) \bigr\vert ^{2}\,\frac{dt}{t} \biggr) ^{1/2}, \\ &\mathcal{N}_{\varOmega,\phi,j}(x,x+1)=\sum_{k\in \mathbf{Z}} \int_{\mathbf{S}^{n-1}}(S _{k+j}f) \bigl(x-ru,x_{n+1}- \phi(r)\bigr)\chi_{{\mathcal{I}_{k,\beta_{\varOmega }}}} \varOmega(u)\,d\sigma(u). \end{aligned}$$

By using [4, ineq. (3.10)] together with Lemma 2.2, we get

2.7 $$ \bigl\Vert \mathcal{T}_{\varOmega,\phi,j}(f) \bigr\Vert _{L^{p}(\mathbf{R}^{n+1})} \leq C_{p,q} \beta_{\varOmega}^{{1/2}} 2^{-\varepsilon_{p} \vert j \vert } \Vert f \Vert _{L^{p}(\mathbf{R}^{n+1})} $$

for some constant $0<\varepsilon_{p}<1$ and for all $2\leq p<\infty$. Therefore, by (2.6) and (2.7), we immediately satisfy inequality (2.5) for all $2\leq p<\infty$. □

## Proof of the main results

### Proof of Theorem 1.1

The proof of Theorem 1.1 mainly depends on the approaches employed in the proof of [11, Theorem 1.1] and [4, Theorem 1.6]. By duality, for $1<\gamma\leq 2$, we get

$$\begin{aligned} \mathcal{M}_{P,\varOmega, \phi}^{(\gamma)}(f) (x,x+1)= \biggl( \int_{0}^{\infty} \biggl\vert \int_{\mathbf{S}^{n-1}}e^{iP(ru)}f\bigl(x-r u,x_{n+1}-\phi(r) \bigr)\varOmega(u)\,d\sigma(u) \biggr\vert ^{\gamma'}\,\frac{dr}{r} \biggr) ^{1/\gamma'}, \end{aligned}$$

which gives

3.1 $$\begin{aligned} \bigl\Vert \mathcal{M}_{P,\varOmega, \phi}^{(\gamma)}(f) \bigr\Vert _{L^{p}(\textbf{R}^{n+1})}= \bigl\Vert N(f) \bigr\Vert _{L^{p}(L^{\gamma'}(\textbf{R}^{+},\frac{dr}{r}),\textbf {R}^{n+1})}, \end{aligned}$$

where $N:L^{p}(\textbf{R}^{n+1})\rightarrow L^{p}(L^{\gamma'}(\textbf{R}^{+},\frac{dr}{r}),\textbf{R}^{n+1})$ is a linear operator defined by

$$\begin{aligned} N(f) (x,x_{n+1},r)= \int_{\mathbf{S}^{n-1}}e^{iP(u)}f\bigl(x-r u,x_{n+1}-\phi(r) \bigr)\varOmega(u)\,d\sigma(u). \end{aligned}$$

Now if we assume that

$$\begin{aligned} \bigl\Vert \mathcal{M}_{P,\varOmega, \phi}^{(2)}(f) \bigr\Vert _{L^{p}(\textbf{R}^{n+1})}= \bigl\Vert N(f) \bigr\Vert _{L^{p}(L^{2}(\textbf{R}^{+},\frac{dr}{r}),\textbf {R}^{n+1})}\leq C_{p,q}(1+\beta_{\varOmega})^{1/2} \Vert f \Vert _{L^{p}( \mathbf{R} ^{n+1})} \end{aligned}$$

for $2\leq p<\infty$; and

$$\begin{aligned} \bigl\Vert \mathcal{M}_{P,\varOmega, \phi}^{(1)}(f) \bigr\Vert _{L^{\infty}(\textbf{R}^{n+1})}= \bigl\Vert N(f) \bigr\Vert _{L^{\infty}(L^{\infty}(\textbf{R}^{+},\frac {dr}{r}),\textbf{R}^{n+1})}\leq C \Vert f \Vert _{L^{\infty}( \mathbf{R} ^{n+1})}, \end{aligned}$$

then by applying the interpolation theorem for the Lebesgue mixed normed spaces to the last two inequalities, we directly obtain

3.2 $$\begin{aligned} \bigl\Vert \mathcal{M}_{P,\varOmega, \phi}^{(\gamma)}(f) \bigr\Vert _{L^{p}(\textbf{R}^{n+1})}\leq C_{p,q}(1+\beta_{\varOmega})^{1/\gamma'} \Vert f \Vert _{L^{p}( \mathbf{R} ^{n+1})} \end{aligned}$$

for $\gamma'\leq p<\infty$ with $1<\gamma\leq2$; and $\Vert \mathcal{M}_{P,\varOmega, \phi}^{(1)}(f) \Vert _{L^{\infty}(\textbf{R}^{n+1})}\leq C \Vert f \Vert _{L^{\infty}( \mathbf{R} ^{n+1})}$. Thus, to prove our theorem, it is enough to prove it only for the cases $\gamma=1$ and $\gamma=2$.

*Case 1* (if $\gamma=1$). Assume that $h\in L^{1}(\textbf{R}^{+},\frac{dr}{r})$ and $f\in L^{\infty}(\textbf{R}^{n+1})$. Then, for all $(x,x_{n+1})\in \textbf{R}^{n}\times\textbf{R}$, we have

$$\begin{aligned} \biggl\vert \int_{0}^{\infty}h(r) \int_{\mathbf{S}^{n-1}}e^{iP(ru)}f\bigl(x-r u,x_{n+1}-\phi(r) \bigr)\varOmega(u))\,d\sigma(u)\,\frac{dr}{r} \biggr\vert \leq C \Vert f \Vert _{ L^{\infty}(\textbf{R}^{n+1})} \Vert h \Vert _{ L^{1}(\textbf{R}^{+},\frac{dr}{r})}. \end{aligned}$$

Hence, by taking the supremum on both sides over all *h* with $\Vert h \Vert _{ L^{1}(\textbf{R}^{+},\frac{dr}{r})}\leq1$, we reach

$$\begin{aligned} \mathcal{M}_{P,\varOmega,\phi}^{(1)}f(x,x_{n+1})\leq C \Vert f \Vert _{ L^{\infty}(\textbf{R}^{n+1})} \end{aligned}$$

for almost every where $(x,x_{n+1})\in\textbf{R}^{n+1}$, which implies

$$\begin{aligned} \bigl\Vert \mathcal{M}_{P,\varOmega ,\phi}^{(1)}f \bigr\Vert _{L^{\infty}(\textbf{R}^{n+1})}\leq C \Vert f \Vert _{ L^{\infty}(\textbf{R}^{n+1})}. \end{aligned}$$

*Case 2* (if $\gamma=2$). We use the induction on the degree of the polynomial *P*. If the degree of *P* is 0, then by Lemma 2.3 we get that, for all $p\geq2$,

3.3 $$\begin{aligned} \bigl\Vert \mathcal{M}^{(2)}_{P,\varOmega,\phi}(f) \bigr\Vert _{L^{p}(\mathbf{ \mathbf{R} }^{n+1})} \leq&C_{p,q} ( 1+\beta_{\varOmega})^{1/2} \Vert f \Vert _{L^{p}( \mathbf{R} ^{n+1})}. \end{aligned}$$

Now, assume that (1.3) is satisfied for any polynomial of degree less than or equal to *m* with $m\geq1$. We need to show that (1.3) is still true if $\operatorname{deg}(P)=m+1$. Let

$$ P(x)=\sum_{|\alpha|\leq m+1}a_{\gamma}x^{\gamma} $$

be a polynomial of degree $m+1$. Without loss of generality, we may assume that $\sum_{ \vert \gamma \vert =m+1} \vert a_{\gamma} \vert =1$, and also we may assume that *P* does not contain $\vert x \vert ^{m+1}$ as one of its terms. Let $\{ \varphi_{k} \}_{k\in\mathbf{Z}} $ be a collection of $\mathcal{C}^{\infty}(0,\infty)$ functions satisfying the following conditions:

$$\begin{aligned} &\operatorname{supp}\varphi_{k} \subseteq\mathcal{I}_{k,\beta_{\varOmega }}= \bigl[ 2^{-(k+1)\beta_{\varOmega}},2^{-(k-1)\beta_{\varOmega}} \bigr];\quad 0\leq\varphi_{k} \leq1; \\ &\sum_{k\in\mathbf{Z}}\varphi_{k} ( r ) =1;\quad \mbox{and} \quad \biggl\vert \,\frac{d^{k}\varphi_{k} ( r ) }{du^{r}} \biggr\vert \leq \frac{C_{k}}{r^{k}}. \end{aligned}$$

Define the multiplier operators $S_{k}$ in $\textbf{R}^{n+1}$ by

$$\begin{aligned} \widehat{{(S_{k}f)}}(\xi,\eta)=\varphi_{k}\bigl( \vert \xi \vert \bigr)\widehat{f}(\xi,\eta)\quad \mbox{for } (\xi,\eta)\in \mathbf{R}^{n}\times\mathbf{R}, \end{aligned}$$

and set

$$ \varGamma_{\infty}(r)=\sum_{k=-\infty}^{0} \varphi_{k}(r), \qquad \varGamma_{0}(r)=\sum _{k=1}^{\infty}\varphi _{k}(r). $$

Thanks to Minkowski’s inequality, we have

3.4 $$ \mathcal{M}^{(2)}_{P,\varOmega,\phi}(f) (x,x_{n+1})\leq \mathcal{M}^{(2)}_{P,\varOmega,\phi,\infty }(f) (x,x_{n+1})+ \mathcal{M}^{(2)}_{P,\varOmega ,\phi,0}(f) (x,x_{n+1}), $$

where

$$\begin{aligned} &\mathcal{M}^{(2)}_{P,\varOmega,\phi,\infty}(f) (x,x_{n+1})\\ &\quad = \biggl( \int_{2^{-\beta_{\varOmega}}}^{\infty} \biggl\vert \varGamma_{\infty}(r) \int_{\mathbf{S}^{n-1}}e^{iP(r u)}f\bigl(x-r u,x_{n+1}-\phi(r) \bigr)\varOmega(u)\,d\sigma(u) \biggr\vert ^{2}\,\frac{dr}{r} \biggr) ^{1/2}, \end{aligned}$$

and

$$\begin{aligned} &\mathcal{M}^{(2)}_{P,\varOmega,\phi,0}(f) (x,x_{n+1})\\ &\quad = \biggl( \int_{0}^{1} \biggl\vert \varGamma _{0}(r) \int_{\mathbf{S} ^{n-1}}e^{iP(r u)}f\bigl(x-ru,x_{n+1}-\phi(r) \bigr)\varOmega(u)\,d\sigma(u) \biggr\vert ^{2}\,\frac{dr}{r} \biggr) ^{1/2}. \end{aligned}$$

Let us first estimate $L^{p}$-norm of $\mathcal{M}^{(2)}_{P,\varOmega ,\phi,\infty}(f)$. Define

$$\begin{aligned} &\mathcal{M}^{(2)}_{P,\varOmega,\phi,\infty,k}(f) (x,x_{n+1})\\ &\quad = \biggl( \int_{2^{-(k+1)\beta_{\varOmega}}}^{2^{-(k-1)\beta_{\varOmega }}} \biggl\vert \int_{\mathbf{S}^{n-1}}e^{iP(r u)}f\bigl(x-r u,x_{n+1}-\phi(r) \bigr)\varOmega(u)\,d\sigma(u) \biggr\vert ^{2}\,\frac{dr}{r} \biggr) ^{1/2}. \end{aligned}$$

Hence, by generalized Minkowski’s inequality, it is easy to show that

3.5 $$\begin{aligned} \mathcal{M}^{(2)}_{P,\varOmega,\phi,\infty}(f) (x,x_{n+1}) \leq &\sum _{k=-\infty}^{0}\mathcal{M}^{(2)}_{P,\varOmega ,\phi,\infty,k}(f) (x,x_{n+1}). \end{aligned}$$

If $p=2$, then by a simple change of variables, Plancherel’s theorem, Fubini’s theorem, and Lemma 2.1, we get that

3.6 $$\begin{aligned} \bigl\Vert \mathcal{M}^{(2)}_{P,\varOmega,\phi,\infty ,k}(f) \bigr\Vert _{L^{2}( \mathbf{R} ^{n+1})} =& \biggl( \int_{ \mathbf{R} ^{n+1}} \bigl\vert \widehat{f}(\zeta,\eta ) \bigr\vert ^{2} \mathcal{J}_{k,\varOmega,\phi}(\zeta,\eta )\,d\zeta \,d\eta \biggr) ^{1/2} \\ \leq &C2^{\frac{(k+1)}{8q'}} (1+\beta_{\varOmega})^{1/2} \Vert f \Vert _{L^{2}( \mathbf{R} ^{n+1})}. \end{aligned}$$

However, if $p>2$, then by the duality, there exists $\varPsi\in L^{(p/2)^{\prime}}( \mathbf{R} ^{n+1})$ with $\Vert \varPsi \Vert _{L^{(p/2)^{\prime}}( \mathbf{R} ^{n+1})}=1$ such that

$$\begin{aligned} & \bigl\Vert \mathcal{M}^{(2)}_{P,\varOmega,\phi,\infty ,k}(f) \bigr\Vert _{L^{p}( \mathbf{R} ^{n+1})}^{2} \\ &\quad = \int_{ \mathbf{R} ^{n+1}} \int_{1}^{2^{2\beta_{\varOmega}}} \biggl\vert \int_{\mathbf{S}^{n-1}}\mathcal{G}_{k,\varOmega,P }(r,u,0,0)f \bigl(x-2^{-(k+1)\beta_{\varOmega}}r u,x_{n+1}\\ &\qquad {}-\phi\bigl(2^{-(k+1)\beta_{\varOmega}}r\bigr) \bigr)\,d\sigma(u) \biggr\vert ^{2}\,\frac{dr}{r} \\ &\qquad {}\times \bigl\vert \varPsi(x,x_{n+1}) \bigr\vert \,dx\,dx_{n+1}. \end{aligned}$$

So, by Hölder’s inequality and Lemma 2.2, we conclude that

$$\begin{aligned} &\bigl\Vert \mathcal{M}^{(2)}_{P,\varOmega,\phi,\infty ,k}(f) \bigr\Vert _{L^{p}( \mathbf{R} ^{n+1})}^{2}\\ &\quad \leq C \int_{\mathbf{R} ^{n+1}} \bigl\vert f(z,z_{n+1}) \bigr\vert ^{2} \int_{1}^{2^{2 \beta_{\varOmega}}} \int_{\mathbf{S}^{n-1}} \bigl\vert \varOmega(u) \bigr\vert \\ &\qquad {}\times \bigl\vert \varPsi\bigl(z+2^{-(k+1)\beta_{\varOmega}}r u,z_{n+1}+\phi \bigl(2^{-(k+1)\beta_{\varOmega}}r\bigr) \bigr) \bigr\vert \,d\sigma (u)\,\frac{dr}{r}\,dz\,dz_{n+1} \\ &\quad \leq C_{p} (1+\beta_{\varOmega}) \bigl\Vert \vert f \vert ^{2} \bigr\Vert _{L^{(p/2)}( \mathbf{R} ^{n+1})} \bigl\Vert \mathcal{M}_{\varOmega,\phi}( \widetilde{\varPsi}) \bigr\Vert _{L^{(p/2)^{\prime}}( \mathbf{R} ^{n+1})} \\ &\quad \leq C_{p} (1+\beta_{\varOmega}) \Vert f \Vert _{L^{p}( \mathbf{R} ^{n+1})}^{2} \Vert \widetilde{\varPsi} \Vert _{L^{(p/2)^{\prime}}( \mathbf{R} ^{n+1})} \Vert \varOmega \Vert _{L^{1}(\mathbf{S}^{n-1})}, \end{aligned}$$

where $\widetilde{\varPsi}(z,z_{n+1})=\varPsi(-z,-z_{n+1})$. Thus,

$$ \bigl\Vert \mathcal{M}^{(2)}_{P,\varOmega,\phi,\infty ,k}(f) \bigr\Vert _{L^{p}( \mathbf{R} ^{n+1})}\leq C_{p} (1+\beta_{\varOmega})^{1/2} \Vert f \Vert _{L^{p}( \mathbf{R} ^{n+1})}, $$

which when combined with (3.6) gives that there is $0<\nu<1$ so that

3.7 $$ \bigl\Vert \mathcal{M}^{(2)}_{P,\varOmega,\phi,\infty ,k}(f) \bigr\Vert _{L^{p}( \mathbf{R} ^{n+1})}\leq C_{p} 2^{\nu (k+1)/8} (1+\beta_{\varOmega})^{1/2} \Vert f \Vert _{L^{p}( \mathbf{R} ^{n+1})} $$

for all $p\geq2$. Therefore, by (3.5) and (3.7), we obtain

3.8 $$ \bigl\Vert \mathcal{M}^{(2)}_{P,\varOmega,\phi,\infty}(f) \bigr\Vert _{L^{p}( \mathbf{R} ^{n+1})}\leq C_{p,q} (1+\beta_{\varOmega})^{1/2} \Vert f \Vert _{L^{p}( \mathbf{R} ^{n+1})}. $$

Now, let us estimate the $L^{p}$-norm of $\mathcal{M}^{(2)}_{P,\varOmega ,\phi,0 }(f)$. Let $Q(x)=\sum_{ \vert \gamma \vert \leq m}a_{\gamma}x^{\gamma}$. Define $\mathcal{M}^{(2)}_{Q,\varOmega ,\phi,0 }(f)$ and $\mathcal{M}^{(2)}_{P,Q,\varOmega,\phi,0 }(f)$ by

$$\begin{aligned} &\mathcal{M}^{(2)}_{Q,\varOmega,\phi,0 }(f) (x,x_{n+1})= \biggl( \int_{0}^{1} \biggl\vert \int_{\mathbf{S} ^{n-1}}e^{iQ(r u)}f\bigl(x-r u,x_{n+1}-\phi(r) \bigr)\varOmega(u)\,d\sigma (u) \biggr\vert ^{2}\,\frac{dr}{r} \biggr) ^{1/2} , \\ &\mathcal{M}^{(2)}_{P,Q,\varOmega,\phi,0 }(f) (x,x_{n+1})\\ &\quad = \biggl( \int_{0}^{1} \biggl\vert \int_{\mathbf{S} ^{n-1}} \bigl(e^{iP(r u)}-e^{iQ(r u)} \bigr)f \bigl(x-r u,x_{n+1}-\phi(r) \bigr)\varOmega(u)\,d\sigma(u) \biggr\vert ^{2}\,\frac{dr}{r} \biggr) ^{1/2}. \end{aligned}$$

Thus, by Minkowski’s inequality, we deduce

3.9 $$ \mathcal{M}^{(2)}_{P,\varOmega,\phi,0 }(f) (x,x_{n+1})\leq \mathcal{M}^{(2)}_{Q,\varOmega,\phi,0 }(f) (x,x_{n+1})+ \mathcal{M}^{(2)}_{P,Q,\varOmega,\phi,0 }(f) (x,x_{n+1}). $$

On the one hand, since $\deg(Q)\leq m$, then by our assumption,

3.10 $$ \bigl\Vert \mathcal{M}^{(2)}_{Q,\varOmega,\phi,0 }(f) \bigr\Vert _{L^{p}( \mathbf{R} ^{n+1})}\leq C_{p,q} (1+\beta_{\varOmega})^{1/2} \Vert f \Vert _{L^{p}( \mathbf{R} ^{n+1})} $$

for all $p\geq2$. On the other hand, since we have

$$\begin{aligned} \bigl\vert e^{iP(r u)}-e^{iQ(r u)} \bigr\vert \leq&r ^{(m+1)} \biggl\vert \sum_{ \vert \gamma \vert =m+1}a_{\gamma}(u)^{\gamma} \biggr\vert \leq r ^{(m+1)}, \end{aligned}$$

then by the Cauchy–Schwarz inequality, we reach that

$$\begin{aligned} &\mathcal{M}^{(2)}_{P,Q,\varOmega,\phi,0 }(f) (x,x_{n+1}) \\ &\quad \leq C \biggl( \int_{0}^{1} \int_{\mathbf{S}^{n-1}} r ^{2(m+1)} \bigl\vert \varOmega(u) \bigr\vert \bigl\vert f\bigl(x-r u,x_{n+1}-\phi(r) \bigr) \bigr\vert ^{2}\,d\sigma(u) \,\frac{dr}{r} \biggr) ^{1/2} \\ &\quad \leq \Biggl( \sum_{j=1 }^{\infty}2^{-j({2(m+1)})} \int_{2^{{-j}}}^{2{-j+1}} \int _{\mathbf{S} ^{n-1}} \bigl\vert \varOmega(u) \bigr\vert \bigl\vert f \bigl(x-ru,x_{n+1}-\phi(r)\bigr) \bigr\vert ^{2}\,d\sigma (u) \,\frac{dr}{r} \Biggr) ^{1/2} \\ &\quad \leq C \bigl( \mathcal{M}_{\varOmega,\phi } \bigl( \vert f \vert ^{2} \bigr) \bigr) ^{1/2}. \end{aligned}$$

Hence, by Lemma 2.2, we get that

3.11 $$\begin{aligned} \bigl\Vert \mathcal{M}^{(2)}_{P,Q,\varOmega,\phi,0 }(f) \bigr\Vert _{L^{p}( \mathbf{R} ^{n+1})} \leq& C_{p} \Vert \varOmega \Vert _{L^{1}(\mathbf{S} ^{n-1})} \bigl\Vert \vert f \vert ^{2} \bigr\Vert _{L^{p/2}( \mathbf{R} ^{n+1})}^{1/2} \\ \leq& C_{p} \Vert f \Vert _{L^{p}( \mathbf{R} ^{n+1})}\leq C_{p} (1+\beta_{\varOmega})^{1/2} \Vert f \Vert _{L^{p}( \mathbf{R} ^{n+1})} \end{aligned}$$

for all $p\geq2$. Therefore, by (3.9)–(3.11), we obtain

3.12 $$ \bigl\Vert \mathcal{M}^{(2)}_{P,\varOmega,\phi,0 }(f) \bigr\Vert _{L^{p}( \mathbf{R} ^{n})}\leq C_{p} (1+\beta_{\varOmega})^{1/2} \Vert f \Vert _{L^{p}( \mathbf{R} ^{n})}. $$

Consequently, by (3.4), (3.8), and (3.12), we finish the proof of Theorem 1.1. □

### Proof of Theorem 1.2

Assume that *Ω* satisfies condition (1.1). If $\varOmega\in L(\log L)^{1/\gamma'}(\mathcal{ \mathbf{S}}^{n-1})$ with $1<\gamma\leq2$, then as in [14], we can decompose *Ω* as a sum of functions in $L^{2}(\mathcal{ \mathbf{S}}^{n-1})$. In fact, we have a sequence $\{\varOmega_{k}:k=0,1,2,\ldots\}$ of functions in $L^{1}(\mathcal{ \mathbf{S}}^{n-1})$ with

$$\varOmega=\sum_{k=0}^{\infty}\varOmega_{k} $$

such that

$$\begin{aligned} & \int_{\mathbf{S}^{n-1}}\varOmega\bigl(x'\bigr) \,d\sigma \bigl(x'\bigr) =0,\qquad \varOmega_{0}\in L^{2}\bigl(\mathcal{ \mathbf{S}}^{n-1}\bigr),\qquad \Vert \varOmega_{k} \Vert _{L^{1}(\mathcal{ \mathbf{S}}^{n-1})}\leq C, \\ & \Vert \varOmega_{k} \Vert _{L^{\infty}(\mathcal{ \mathbf{S}}^{n-1})}\leq C 2^{4k},\quad \mbox{and}\quad \varOmega=\sum_{k=1}^{\infty}k^{1/\gamma'} \Vert \varOmega_{k} \Vert _{L^{1}(\mathcal{ \mathbf{S}}^{n-1})}\leq C \Vert \varOmega \Vert _{L(\log L)^{1/\gamma'}(\mathcal{ \mathbf{S}}^{n-1})}. \end{aligned}$$

Thus, we get the following:

3.13 $$\begin{aligned} \mathcal{M}_{P,\varOmega, \phi}^{(\gamma)}(f) (x,x+1) \leq&\mathcal{M}_{P,\varOmega_{0}, \phi}^{(\gamma)}(f) (x,x+1) \\ &{}+\sum_{k=1}^{\infty} \Vert \varOmega_{k} \Vert _{L^{1}(\mathcal{\mathbf{S}}^{n-1})} \mathcal{M}_{P,\varOmega_{k}, \phi}^{(\gamma)}(f) (x,x+1). \end{aligned}$$

Since $\varOmega_{0}\in L^{2}(\mathcal{\mathbf{S}}^{n-1})$, then we have

3.14 $$ \bigl\Vert \mathcal{M}_{P,\varOmega_{0} ,\phi}^{(\gamma)}(f) \bigr\Vert _{L^{p}( \mathbf{R} ^{n+1})}\leq C_{p} \bigl(1+\log^{1/\gamma'} \bigl(e+ \Vert \varOmega_{0} \Vert _{L^{2}(\mathbf{S}^{n-1})}\bigr)\bigr) ) \Vert f \Vert _{L^{p}( \mathbf{R} ^{n+1})} $$

for $\gamma'\leq p<\infty$, and since

3.15 $$ \bigl(1+\log^{1/\gamma'} \bigl(e+ \Vert \varOmega_{k} \Vert _{L^{\infty}(\mathbf{S}^{n-1})}\bigr) \bigr)\leq \bigl(1+\log^{1/\gamma'} \bigl(e+C2^{4k}\bigr) \bigr)\leq Ck^{{1/\gamma'}}, $$

then by Minkowski’s inequality and (3.13)–(3.15), we deduce that

$$\begin{aligned} \bigl\Vert \mathcal{M}_{P,\varOmega,\phi}^{(\gamma)}(f) \bigr\Vert _{L^{p}( \mathbf{R} ^{n+1})} \leq& \bigl\Vert \mathcal{M}_{P,\varOmega_{0} ,\phi}^{(\gamma)}(f) \bigr\Vert _{L^{p}( \mathbf{R} ^{n+1})}+\sum_{k=1}^{\infty} \Vert \varOmega_{k} \Vert _{L^{1}(\mathcal{ \mathbf{S}}^{n-1})} \bigl\Vert \mathcal{M}_{P,\varOmega_{k} ,\phi}^{(\gamma)}(f) \bigr\Vert _{L^{p}( \mathbf{R} ^{n+1})} \\ \leq&C_{p} \Biggl(1+\sum_{k=1}^{\infty} \Vert \varOmega_{k} \Vert _{L^{1}(\mathcal{ \mathbf{S}}^{n-1})}k^{{1/\gamma '}} \Biggr) \Vert f \Vert _{L^{p}( \mathbf{R} ^{n+1})} \\ \leq&C_{p} \Vert \varOmega \Vert _{L(\log L)^{1/\gamma'}(\mathcal{ \mathbf{S}}^{n-1})} \Vert f \Vert _{L^{p}( \mathbf{R} ^{n+1})}\leq C_{p} \Vert f \Vert _{L^{p}( \mathbf{R} ^{n+1})}. \end{aligned}$$

However, if $\varOmega\in B_{q}^{(0,-1/\gamma)}( \mathcal{\mathbf{S}}^{n-1})$ with $q>1$ and $1<\gamma\leq2$, then

$$\varOmega=\sum_{\mu=1}^{\infty}c_{\mu}b_{\mu}, $$

where each $c_{\mu}$ is a complex number, each $b_{\mu}$ is a *q*-block supported in an interval $I_{\mu}$ on $( \mathcal{\mathbf{S}}^{n-1})$ and

3.16 $$ M_{q}^{(0,-1/\gamma)}\bigl(\{c_{\mu}\}\bigr)=\sum _{\mu=1}^{\infty} \vert c_{\mu} \vert \bigl( 1+\log^{{1/\gamma'}} \bigl( \vert I_{\mu} \vert ^{-1} \bigr) \bigr)< \infty. $$

For each *μ*, define the blocklike function $\widetilde{b_{\mu}}$ by

3.17 $$ \widetilde{b_{\mu}}(x)=b_{\mu}(x)- \int_{\mathbf{S}^{n-1}}{b_{\mu}} (y) \,d\sigma(y). $$

Then it is easy to show that $\widetilde{b_{\mu}}(x)$ has the following properties:

$$\begin{aligned} \int_{\mathbf{S}^{n-1}}\widetilde{b_{\mu}} (y) \,d\sigma(y)=0,\qquad \Vert \widetilde{b_{\mu}} \Vert _{L^{1}(\mathcal{ \mathbf{S}}^{n-1})}\leq C\quad \mbox{and} \quad \Vert \widetilde{b_{\mu}} \Vert _{L^{q}(\mathcal{ \mathbf{S}}^{n-1})}\leq C \vert I_{\mu} \vert ^{-1/q'}. \end{aligned}$$

Without loss of generality, we may assume that $\vert I_{\mu} \vert <1$. So,

3.18 $$ \mathcal{M}_{P,\varOmega, \phi}^{(\gamma)}(f) (x,x+1)\leq\sum _{\mu=1}^{\infty} \vert c_{\mu} \vert \mathcal{M}_{P,\widetilde{b_{\mu}}, \phi}^{(\gamma)}(f) (x,x+1). $$

Therefore, by Minkowski’s inequality and the above procedure, we get that

$$\begin{aligned} \bigl\Vert \mathcal{M}_{P,\varOmega,\phi}^{(\gamma)}(f) \bigr\Vert _{L^{p}( \mathbf{R} ^{n+1})} \leq& C_{p,q} \sum_{\mu=1}^{\infty} \vert c_{\mu} \vert \bigl(1+\log^{1/\gamma'}\bigl(e+ \vert I_{\mu} \vert ^{-1} \bigr) \bigr) \Vert f \Vert _{L^{p}( \mathbf{R} ^{n+1})} \\ \leq& C_{p,q} \Vert f \Vert _{L^{p}( \mathbf{R} ^{n+1})} \end{aligned}$$

for all $p\geq\gamma'$. □

## Further results

In this section, we present some additional results that follow by applying Theorems 1.1 and 1.2. The first result concerns the boundedness of oscillatory singular integrals. More precisely, we deduce the following.

### Theorem 4.1

*Assume that* $\varOmega\in L(\log L)^{1/\gamma'}(\mathcal{ \mathbf{S}}^{n-1})\cup B_{q}^{(0,-1/\gamma)}( \mathcal{\mathbf{S}}^{n-1})$, $q>1$ *and satisfying condition* (1.1). *Let* $h\in\mathfrak{L}^{\gamma}( \mathbf{R} ^{+})$ *for some* $1<\gamma\leq2$ *and* *ϕ* *be given as in Theorem *1.1. *Then the singular integral operator* $T^{(\gamma)}_{P,\varOmega, h,\phi}$ *given by*

4.1 $$ T^{(\gamma)}_{P,\varOmega, h,\phi}(f) (x,x_{n+1})=p\cdot v \int_{ \mathbf{R} ^{n}}e^{iP(y)}f\bigl(x-y,x_{n+1}-\phi\bigl( \vert y \vert \bigr) \bigr)K_{\varOmega,h}(y)\,dy $$

*is bounded on* $L^{p}(\textbf{R}^{n+1})$ *for* $1< p<\infty$.

### Proof

The proof of this case is reached by using the observation that

4.2 $$ \bigl\vert T^{(\gamma)}_{P,\varOmega, h,\phi}(f) (x,x+1) \bigr\vert \leq \Vert h \Vert _{L^{\gamma}( \mathbf{R} ^{+},\frac{dr}{r})} \mathcal{M}_{P,\varOmega, \phi}^{(\gamma)}(f) (x,x+1). $$

In fact, by the last inequality and Theorem 1.2, we obtain that $T^{(\gamma)}_{P,\varOmega, h,\phi}$ is bounded on $L^{p}(\textbf{R}^{n+1})$ for $\gamma'\leq p<\infty$ with $1<\gamma\leq2$. Furthermore, by a standard duality argument, we satisfy the $L^{p}$ boundedness of $T^{(\gamma)}_{P,\varOmega, h,\phi}$ for $1< p\leq\gamma$ with $1<\gamma\leq2$. So, if $\gamma=2$, then we are done. However, if $1<\gamma<2$, then we apply the real interpolation theorem to attain the $L^{p}$ boundedness of $T^{(\gamma)}_{P,\varOmega, h,\phi}$ for ($\gamma< p<\gamma'$). This completes the proof. □

The generalized parametric Marcinkiewicz operator related to the operator $\mathcal{M}_{P,\varOmega, \phi}^{(\gamma)}$ is defined by

4.3 $$\begin{aligned} &{\mu}_{P,\varOmega, \phi}^{(\gamma)}(f) (x,x+1) \\ &\quad = \biggl( \int _{\textbf{R}^{+}} \biggl\vert \frac{1}{t} \int_{ \vert y \vert \leq t}e^{iP(y)}f\bigl(x-y,x_{n+1}-\phi \bigl( \vert y \vert \bigr)\bigr) \varOmega(y) { \vert y \vert ^{-n+1}}\,dy \biggr\vert ^{\gamma'}\,\frac{dt}{t} \biggr)^{1/\gamma'}. \end{aligned}$$

As a direct consequence of the notice that

$$ {\mu}_{P,\varOmega, \phi}^{(\gamma)}(f) (x,x+1)\leq C \mathcal{M}_{P,\varOmega, \phi}^{(\gamma)}(f) (x,x+1) $$

for $1\leq\gamma\leq2$, it is easy to derive the following result.

### Theorem 4.2

*Let* *Ω* *satisfy condition* (1.1) *and belong to the space* $L(\log L)^{1/\gamma'}(\mathcal{ \mathbf{S}}^{n-1})\cup B_{q}^{(0,-1/\gamma)}( \mathcal{\mathbf{S}}^{n-1})$ *for some* $q>1$ *and* $1\leq\gamma\leq2$. *Suppose that* *ϕ* *and* *P* *are given as in Theorem *1.2. *Then the parametric Marcinkiewicz operator* ${\mu}_{P,\varOmega, \phi}^{(\gamma)}$ *is bounded on* $L^{p}(\textbf{R}^{n+1})$ *for* $\gamma'\leq p<\infty$ *with* $1<\gamma\leq2$; *and it is bounded on* $L^{\infty}(\textbf{R}^{n+1})$ *for* $\gamma=1$.

We point out that by specializing to the case $P=0$, $\gamma=2$ and $\phi(t)=t$, then the operator ${\mu}_{P,\varOmega, \phi}^{(\gamma)}$ (denoted by ${\mu}_{\varOmega}$) is just the classical Marcinkiewicz integral operator introduced by Stain in [29] in which he showed that ${\mu}_{\varOmega}$ is of type $(p,p)$ for $1 < p\leq2$ provided that $\varOmega\in \mathrm{Lip}_{\alpha}(\mathbf{S}^{n-1})$ for some $0<\alpha\leq2$. Subsequently, the operator ${\mu}_{\varOmega}$ has been studied by many authors (for instance, see [11, 13, 15, 18], as well as [19] and the references therein). For the significance and recent advances on the study of the generalized parametric Marcinkiewicz operators, we refer the readers to consult [7] and [6] among others.

It is worth mentioning that Theorem 4.1 generalizes the corresponding results in [4, 14, 16], and [22]. However, Theorem 4.2 extends and improves the results found in [11, 13, 19], and [29].
